# Patient with congenital factor VII deficiency undergoing brain tumor neurosurgery successfully treated with recombinant factor VIIa and fresh frozen plasma: A case report and literature review

**DOI:** 10.1097/MD.0000000000036694

**Published:** 2023-12-29

**Authors:** Chaoyu Huang, Yongjia Yu, Ningneng Zhai, Wuning Mo, Faquan Lin

**Affiliations:** a Department of Clinical Laboratory, The First Affiliated Hospital of Guangxi Medical University, Key Laboratory of Clinical Laboratory Medicine of Guangxi Department of Education, Nanning, Guangxi, China; b Department of Neurosurgery, The First Affiliated Hospital of Guangxi Medical University, Nanning, Guangxi, China.

**Keywords:** brain tumor, factor VII deficiency, neurosurgery, recombinant factor VIIa, surgical hemostasis

## Abstract

**Rationale:**

Congenital factor VII deficiency is the most common among rare bleeding disorders, characterized by spontaneous or traumatic bleeding. The clinical manifestation is heterogeneous, ranging from asymptomatic phenotype to life-threatening hemorrhages. Intracranial hemorrhage is a common complication of brain tumor neurosurgery, which significantly challenges the perioperative management of patients with hemostatic defects.

**Patient concerns:**

This report presented a 55-year-old man with congenital factor VII deficiency, who had no history of hemorrhage or family history. He underwent a craniotomy for the treatment of papillary craniopharyngioma.

**Diagnoses:**

The patient was diagnosed as papillary craniopharyngioma, factor VII deficiency, and atrial fibrillation.

**Interventions:**

To prevent bleeding, a total of 8 doses of recombinant activated factor VII and 1 dose of fresh frozen plasma were administered as the perioperative replacement therapy. This scheme was guided by a pharmacodynamic evaluation, laboratory tests, and imaging examinations.

**Outcomes:**

No excessive surgical bleeding was observed during the 22-day treatment. The patient was found to have compound heterozygous mutations, Ala304Thr (c.910G > A) and IVS5-2A > G (c.572-2A > G), in the F7 gene.

**Lessons:**

This is the first reported case in which surgical hemorrhage secondary to brain tumor resection was successfully controlled in the presence of congenital factor VII deficiency. Perioperative coagulation state, hemostasis, and thrombosis events should be closely observed, and the interval and dosage of recombinant factor VIIa should be adjusted accordingly.

## 1. Introduction

Congenital factor VII deficiency (FVIID), caused by mutations in the *F7* gene, is a rare autosomal recessive blood disorder with an estimated prevalence of 1:500,000.^[1–3]^ FVIID can cause spontaneous bleeding, including menorrhagia, ecchymosis, epistaxis, gum bleeding, hematuria, gastrointestinal bleeding, hemarthrosis, and intracranial hemorrhage.^[4]^ Unlike hemophilia, factor VII activity (FVII:C) does not serve as a predictor of hemorrhage severity.^[5]^ The primary therapeutic option is replacement therapy (RT), which includes recombinant activated factor VII (rFVIIa), fresh frozen plasma (FFP), plasma-derived factor VII, and prothrombin complex concentrates.^[6]^ Furthermore, 40% to 70% of patients remain asymptomatic,^[4,7,8]^ for whom surgery is considered a major threat.^[9]^ Most of them are occasionally diagnosed by a prolonged prothrombin time (PT) and a decreased FVII:C in preoperative coagulation tests.^[10]^ Despite the absence of previous bleeding episodes, the inherent hemostatic defect is consistently a predisposition to excessive bleeding. In patients with coagulation factor deficiency, the prevention of surgical bleeding should be meticulously considered.^[11]^ However, effective guidelines for the perioperative coagulation management of patients with FVIID have yet to be established, and consensus on the optimal strategy for rFVIIa infusion remains elusive.^[12]^ Currently, the perioperative management of patients with FVIID are individualized, considering factors such as hemorrhage history, family history, FVII:C baseline, and the specific type of surgery.^[10]^

Craniotomy is a significantly risky procedure compared to general surgical procedures. Whether it is for trauma, cerebrovascular disease, or tumor, intracranial hemorrhage is a common complication with considerable rates of disability and mortality. The prevention and management of perioperative intracranial hemorrhage are major concerns for neurosurgeons. However, cases of FVIID involving craniotomy for treatment of non-hemorrhagic disorder are insufficient in the available literature, and surgical treatment for brain tumor in patient with FVIID has not been reported.

In this report, we present an asymptomatic FVIID patient, who underwent a craniotomy for papillary craniopharyngioma resection. In addition, similar cases managed with rFVIIa were reviewed. This is the first report on perioperative management of brain tumor resection in patient with FVIID. This case serves an example that, repeated rFVIIa infusions can effectively prevent and control intracranial hemorrhage complicated by craniotomy for brain tumor resection.

## 2. Case presentation

A 55-year-old man suffered from recurrent headache, dizziness, and progressively blurred vision for over 2 months. To clarify diagnosis and treatment, he went to a local hospital for medical consultation. Magnetic resonance imaging revealed a space-occupying lesion in the sellar region. After examinations, he was diagnosed as benign tumor in the sellar region, along with optic nerve compression, factor VII deficiency, and atrial fibrillation. Considering the significant risk of craniotomy, the hospital recommended transferring him to a superior hospital. One week after his discharge, the patient came to our hospital for further treatment.

**Figure 1.**
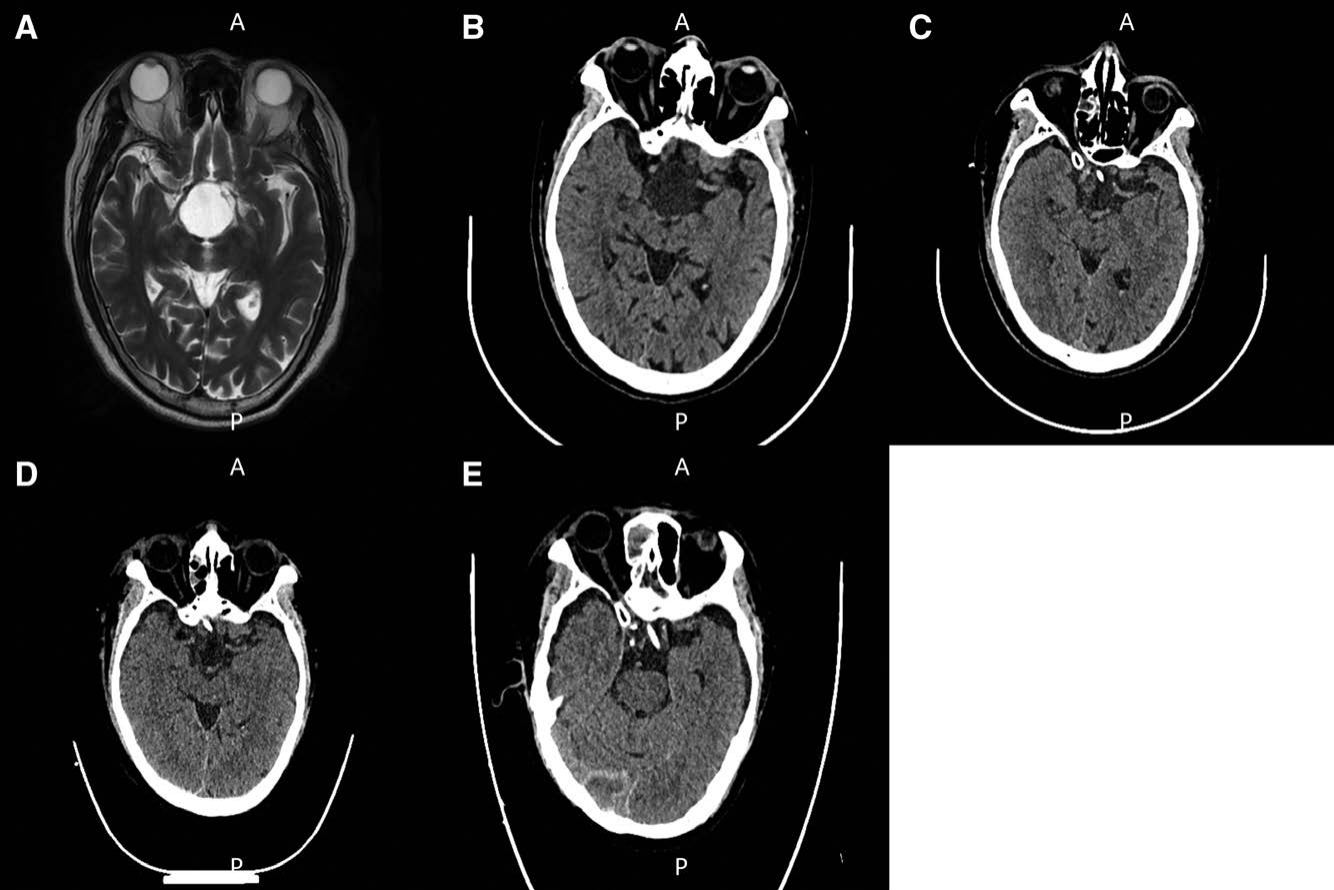
Cerebral CT and MRI results of the patient. (A) Cranial MRI and (B) CT showed cystic masses in the super sellar region. (C) Cranial CT showed scattered small patchy hematocele in the surgical area 6 hours after the surgery. (D) The density of hematocele decreased 3 days after the surgery. (E) Intracranial hematocele was resolved 6 days after the surgery. CT = computed tomography, MRI = magnetic resonance imaging.

Upon admission, the patient denied personal and family history of spontaneous hemorrhage. The physical examination showed: body temperature 36.0°C, pulse rate 55 times/min, respiratory rate 20 times/min, blood pressure 105/81 mm Hg, body weight 74 kg. He was neurologically normal but complained of headache, dizziness, and vision decrease, and his heart rhythm was normal. A risk assessment of venous thromboembolism was conducted, which indicated low risk with a Caprini score of 1. Laboratory examination showed normal hepatic and renal function but an isolated prolonged PT of 43.60 seconds and a decreased FVII:C of 2.0% (Table 1). For full workup, computed tomography (CT), cerebral volume scanning, magnetic resonance imaging (1.5 T) plain scanning and enhanced scanning were performed. The imaging examination showed cystic masses in the suprasellar region and calcification lesions in the right basal ganglia area (Fig. 1A and B). Echocardiography showed enlargement of left and right atrium, moderate mitral regurgitation, and slight tricuspid valve regurgitation.

**Table 1:** Laboratory tests results of the patient before treatment.

Test	Value	Unit	Abbreviation
*Coagulation pathway*			
Prothrombin time	43.60	s	PT
International normalized ratio	3.93		INR
Prothrombin activity	17	%	PTA
Fibrinogen	3.13	g/L	FIB
Activated partial thromboplastin time	33.50	s	APTT
Thrombin time	11.70	s	TT
*Coagulation factors activity*			
Factor VIII activity	96.0	%	FVIII:C
Factor IX activity	101.6	%	FIX:C
Factor XI activity	77.1	%	FXI:C
Factor V activity	97.2	%	FV:C
Factor XII activity	44.4	%	FXII:C
Factor VII activity	2.0	%	FVII:C
Factor II activity	84.1	%	FII:C
Factor X activity	84.5	%	FX:C
*Thromboelastogram*			
Reaction time	6.40	min	R
α angle	70.00	degree	ANGLE
Maximum amplitude	56.90	mm	MA
Estimate percent lysis	0.00		EPL
Percent lysis of the clot at 30 minutes	0.00		LY30
K time	1.4	min	K
Amplitude	57.5	mm	A
Elastic shear modulus	6600.00	dynes/cm^2^	G
Clotting index	−0.20		CI

Subsequently, his case was further discussed at the multidisciplinary team (MDT) meeting, which included 7 neurosurgeons, a hematologist, an endocrinologist, a laboratory physician, and an anesthesiologist. The neurosurgeons suggest that the tumor growth would further compress the optic nerve, hypophysis, hypothalamus and cerebrospinal fluid circulation, and encompass the carotid artery, thereby leading to vision loss, hypopituitarism, and life-threatening hydrocephalus. The hematologist and laboratory physician highlighted that, the patient had FVIID and atrial fibrillation for years, both of which simultaneously carried the risks of surgical bleeding and thrombosis. Despite the absence of bleeding phenotypes, the FVII:C levels should be maintained above 20% through preoperative RTs. Given these conditions, the MDT decided to offer him surgical treatment and preoperative coagulation correction along with intensive monitoring of coagulation function, hemorrhage, thrombosis and other potential complications. The patient and his family agreed to use rFVIIa for RT.

**Figure 2.**
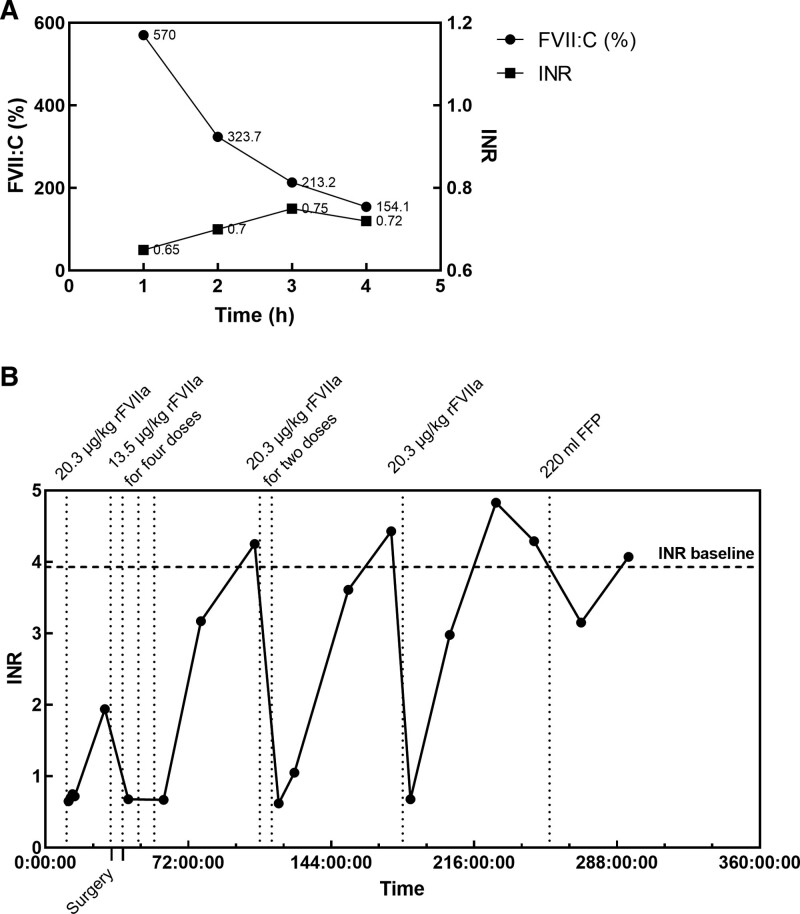
Dynamics changes of coagulation function test results. (A) Plasma FVII activity and international normalized ratio within 4 hours after the first dose. (B) International normalized ratio. Time axis starts from 0:00 am on the 8th hospitalization day.

Preoperatively, the pharmacodynamics of rFVIIa was evaluated through hourly measurement of FVII:C levels, thromboelastograms, and PTs and international normalized ratio (INR) following an intravenous administration of 1.5 mg rFVIIa, equivalent to 20.3 μg/kg body weight (Fig. 2A and Table 2). At 9:18 am the next day, a craniotomy was performed for the excision of the tumor in the supersellar region. Thirty minutes before the surgery began, a dose of 1.0 mg rFVIIa, equivalent to 13.5 μg/kg, was given for the prophylaxis against intraoperative bleedings. Intraoperatively, upon opening the skull, the surface of dura bled and was treated with bipolar electrocoagulation. To prevent excessive bleeding, rFVIIa was used again at the same dosage. The tumor was resected incompletely due to the adhesion of the tumor capsule to the third ventricle and hypothalamus. The operation was successful and lasted for 347 minutes, with bleeding amount of approximately 300 mL. No red blood cell or plasma were infused. The tumor was diagnosed as papillary craniopharyngioma (World Health Organization Grade 1) by histopathology, supported by antigen staining and Periodic Acid-Schiff staining.

**Table 2:** Perioperative management and coagulation function test results during the hospitalization.

Hospitalization day and time*	Coagulation pathway			Coagulation factors activity				Thromboelastogram					
	PT (s)	INR	APTT (s)	FV:C (%)	FVII:C (%)	FII:C (%)	FX:C (%)	R (min)	ANGLE (degree)	MA (mm)	LY30	K (min)	CI
Day 2 6:14	43.6	3.93	33.5	97.2	2.0	84.1	84.5	6.4	70.0	56.9	0.0	1.4	−0.2
Day 8 10:40–20.3 μg/kg rFVIIa													
Day 8 11:40	6.0	0.65	32.1	201.7	570.0	226.3	180.4	4.0	71.4	65.8	0.1	1.3	2.6
Day 8 12:40	6.6	0.70	28.9	190.7	323.7	199.7	148.9	3.8	74.4	58.7	0.1	1.1	2.1
Day 8 13:40	7.5	0.75	30.0	149.4	213.2	162.4	128.3	4.8	70.5	65.1	0.1	1.3	1.9
Day 8 14:44	8.0	0.72	31.2	145.5	154.1	153.4	114.6	5.3	54.4	73.3	0.1	1.2	1.4
Day 9 5:58	21.5	1.94	33.2	–	–	–	–	–	–	–	–	–	–
Day 9 8:48–13.5 μg/kg rFVIIa													
Day 9 9:18—beginning of the craniotomy													
Day 9 14:48–13.5 μg/kg rFVIIa													
Day 9 15:07—end of the craniotomy													
Day 9 17:45	8.0	0.68	24.7	253.9	220.0	249.5	227.4	–	–	–	–	–	–
Day 9 22:48–13.5 μg/kg rFVIIa													
Day 10 6:48–13.5 μg/kg rFVIIa													
Day 10 11:33	7.8	0.67	30.9	–	–	–	–	–	–	–	–	–	–
Day 11 6:23	35.2	3.17	30.8	–	–	–	–	–	–	–	–	–	–
Day 12 9:26	47.2	4.25	31.7	92.6	2.6	80.0	82.8	–	–	–	–	–	–
Day 12 12:00–20.3 μg/kg rFVIIa													
Day 12 18:00–20.3 μg/kg rFVIIa													
Day 12 21:32	7.2	0.62	27.8	–	–	–	–	–	–	–	–	–	–
Day 13 5:24	11.7	1.05	28.9	–	–	–	–	–	–	–	–	–	–
Day 14 8:40	40.1	3.61	31.6	–	–	–	–	–	–	–	–	–	–
Day 15 6:16	49.2	4.43	32.5	–	–	–	–	–	–	–	–	–	–
Day 15 12:00–20.3 μg/kg rFVIIa													
Day 15 15:57	8.0	0.68	28.0	–	–	–	–	–	–	–	–	–	–
Day 16 11:47	33.1	2.98	33.6	–	–	–	–	6.7	77.8	61.6	0.1	0.9	1.0
Day 17 11:00	53.6	4.83	31.2	–	–	–	–	–	–	–	–	–	–
Day 18 6:16	47.6	4.29	31.2	–	–	–	–	–	–	–	–	–	–
Day 18 14:00–220 ml FFP													
Day 19 5:57	35.0	3.15	31.3	–	–	–	–	–	–	–	–	–	–
Day 20 5:49	45.2	4.07	29.8	–	–	–	–	–	–	–	–	–	–

* Day 1 represents the day the patient was admitted.

After the surgery, a risk assessment of venous thromboembolism was conducted, where the Caprini score is 6, indicating high risk. Cranial CT showed a small amount of bleeding in the surgical area (Fig. 1C). Consequently, 2 doses of rFVIIa (13.5 μg/kg) was used to prevent excessive postoperative bleeding. The following morning, the patient had regained consciousness from anesthesia. He reported pain at the surgical site, without any other significant discomfort. The intervals of FVII supplementation were extended, and the patient’s coagulation function was monitored by daily measurements of PT and INR (Fig. 2B and Table 2). Three days after the surgery, the patient was found drowsy and slow to response. A CT scan showed a minor hematocele in the surgical region (Fig. 1D). The INR level had elevated to the baseline (Fig. 2B and Table 2). Consequently, 2 doses of rFVIIa (20.3 μg/kg) was given to him. Six days after the surgery, his consciousness had recovered well. The follow-up cranial CTs revealed that no new hemorrhage occurred, and the intracranial hematocele was resolved (Fig. 1D and E). Thrombosis in the heart and lower limb veins was ruled out by ultrasounds. After 12 days of postoperative observation, the patient recovered well, without hemorrhage, thrombosis or arrhythmia, and was discharged on the 22-nd day of hospitalization.

The sequencing finally revealed Ala304Thr (c.910G>A) and IVS5-2A>G (c.572-2A>G) compound heterozygous mutations in *F7* gene. No hemostatic and antithrombotic drugs were used.

## 3. Review and discussion

Perioperative bleeding is a potential complication of surgical procedures, which is easily triggered in FVIID due to the presence of hemostatic defect.^[9,13]^ Surgical bleeding is not uncommon in the setting of FVIID. Approximately 17% to 30% of cases will experience surgical bleeding, and the prevalence is about 8% in the asymptomatic cases.^[7,14]^ An MDT discussion including hematologists is required for the surgical treatment of patients with FVIID.^[13]^ RT is routinely used in the perioperative management of FVIID, with rFVIIa being the most commonly used (78%), followed by FFP (10%), plasma-derived factor VII concentrate (10%), and prothrombin complex concentrates (2%).^[3]^ rFVIIa has been widely used in the United States and Europe. Generally, it is recommended to dose 15 to 30 μg/kg of rFVIIa every 4 to 6 hours until the surgical incision heals and hemostasis is achieved for patients with plasma FVII:C < 20% undergoing major surgery.^[15,16]^ However, factors other than baseline FVII:C level may affect hemorrhagic outcomes. Spontaneous bleeding occurred previously has been shown to be significantly associated with surgical bleeding.^[14]^ Therefore, it is important to individualize the dosage and interval of RTs for each patient. Sheth et al^[12]^ proposed an algorithm for perioperative management, where a FVII:C threshold of 20% was used to distinguish risk of bleeding, while hemorrhage history, family history and whether to undergo major surgery were taken into account. The risk of surgical bleeding should be more conservatively evaluated in children. Benlakhal et al^[14]^ suggested that for prepubertal patients with FVII:C levels of 10% to 30%, RT should be given regardless of whether they had a hemorrhage history, as they had almost never encountered the hemostasis challenge, which makes the negative hemorrhage history less reliable than that of adults. These aforementioned studies collectively reflected that personal and family hemorrhage history is important in predicting surgical bleeding.

The rFVIIa regimen for patients with FVIID have been evaluated in prospective observational studies. Mariani et al study suggested that a regimen of 3 doses, with the first dose exceeding 13 μg/kg on the operative day, is capable to effectively prevent bleeding during major surgery.^[13]^ Another study by Di Minno et al revealed that a history of major bleeding, rather than plasma FVII levels, was the sole independent predictor of the number of RT doses and RT duration.^[10]^ Interestingly, asymptomatic patients undergoing major surgery received RT at an average of moderate doses but for just 1 day, most likely limited to the operative day, which is remarkably less than the recommended scheme.^[10]^ Similar low-dose rFVIIa RTs in the context of asymptomatic FVIID, major surgery and FVII:C < 5% were also described in some case reports. These include one case with only 2 doses at 15 μg/kg^[17]^ and 3 cases with only single doses within 6 μg/kg.^[18]^ Khazi et al also reported one patient who did not receive any RT.^[19]^ However, several cases receiving remarkably more rFVIIa doses were also reported,^[17,20,21]^ and these may represent unusual cases, suggesting that the dosage of rFVIIa may also be influenced by the type of surgery, clinical pre-thrombotic status, anticoagulant drugs, and the experience of clinical physicians (Table 3). Overall, patients with asymptomatic FVIID undergoing major surgery tend to receive lower dosage of rFVIIa than the recommended dosage, and perioperative management of unusual cases should be orchestrated under the thorough consideration of special surgical complications, thrombosis risk, and medication history.

**Table 3:** Replacement therapy with rFVIIa on preoperative days/operative day/postoperative days in asymptomatic patients who underwent major surgery.

RT day*	Administrations*	Total dose* (μg/kg)	Daily dose* (μg/kg)	Single dose† (μg/kg)	Surgical procedure	Baseline FVII:C	Additional medical history	Reference
1/1/9	1/3/4 (one FFP additionally)	20.3/40.5/74.3	20.3/40.5/8.3	16.9 (13.5–20.3)	Brain tumor craniotomy	2.0%	Atrial fibrillation	This report
0/1/2	0/2/3	0/40/60	0/40/30	20 (20–20)	Cavernous angioma neurosurgery	8%	Appendectomy without any complication	^[22]^
0/1/3	0/2/3	0/22/21	0/22/7	7 (7–15)	Coronary artery bypass graft	8%	Perioperative anticoagulation with UH and LMWH	^[17]^
0/1/0	0/2/0	0/30/0	0/30/0	15 (15–15)	Laparoscopic cholecystectomy	1.4%	–	^[23]^
0/1/0	0/1/0	0/6/0	0/6/0	6	Total thyroidectomy	<1%	Postoperative antifibrinolysis with TXA	^[18]^
0/1/0	0/1/0	0/3.3/0	0/3.3/0	3.3	Total knee replacement	4%	Postoperative anticoagulation with LMWH	^[18]^
0/1/0	0/1/0	0/3/0	0/3/0	3	Laparoscopic bilateral repair of inguinal hernia	5%	–	^[18]^
–	0	0	–	–	Coronary artery bypass	15.2%	AMI, anticoagulation with heparin	^[19]^
0/1/9	0/3/13	0/54.0/234.2	0/54.0/26.0	18 (18–18)	Shoulder arthroscopy	8%	Surgery complicated by excessive bleeding	^[20]^
0/1/9	0/3/13	0/50/162.5	0/50.0/18.0	12.5 (12.5–25)	Knee arthroscopy	8%	Several surgical procedures under cover of RTs	^[20]^
0/1/3	0/4/12	0/75.5/226.4	0/75.5/75.5	18.9 (18.9–18.9)	Modified radical hysterectomy	<1%	Three uneventful vaginal deliveries	^[21]^
1/1/4	Continuous infusion	0.6/0.6/1.8‡	0.6/0.6/0.45‡	–	Cavernous angioma neurosurgery	10%	–	^[24]^
0/1/5	0/1/2 (continuous infusion additionally)	0/20/210‡	0/20/42‡	20 (20–20)	Lobectomy of lung	31%	–	^[25]^

AMI = acute myocardial infarction, LMWH = low molecular weight heparin, RT = replacement treatment, TXA = tranexamic acid, UH = unfractionated heparin.

* Show values on preoperative days/operative day/postoperative days.

† Values given as median (range).

‡ Values were estimated since the exact data was not available.

Our patient had no history of hemorrhage or family history. He was scheduled for craniotomy, and his baseline FVII:C level was 2.0%. Bleeding in the surgical region might lead to a second craniotomy treatment, or even disability or mortality. Given these, the low doses of rFVIIa similar to the reports mentioned above are deem inappropriate for our case. While rFVIIa has been commonly used for hemostasis in neurosurgery in patients without FVIID, the regimen may not be suitable for neurosurgery in patients with FVIID.^[26]^ Therefore, we opted to first evaluate the pharmacokinetics of 1.5 mg FVIIa (20.3 μg/kg), and subsequently adjusted the dosage of rFVIIa based on the evaluation result, administering it at the recommended intervals. Twenty-four hours after the surgery, we extended the intervals between administrations, regarding the high incidence of venous thrombosis in brain tumor surgery.^[27]^ Doses were given only when PT and INR returned to the baselines, thereby preventing hypercoagulability (Table 2). Meanwhile, the atrial fibrillation-related cardiac dysfunction and thrombosis were closely monitored.

Postoperative thrombosis events are infrequent but not absent in FVIID.^[28–30]^ Importantly, FVIID is not a protective factor for postoperative thrombosis.^[28]^ Factors that influence the risk of postoperative thrombosis in patients with FVIID include type of RT, prethrombotic status before surgery, and type of surgery.^[31,32]^ The patient’s craniopharyngioma had the potential to invade the hypothalamus and pituitary gland, which may result in complex postoperative complications involving electrolyte disturbance, endocrine disturbance, and subsequent hormone RT.^[33,34]^ These factors could potentially increase the risk of venous thrombosis. Additionally, the patient had atrial fibrillation, known to increase the risk of thrombosis. His atrial fibrillation was asymptomatic and untreated for many years. The use of anticoagulants may lead to a failure of intracranial hemostasis. In view of these considerations, we opted not to use anticoagulants and instead closely monitored the patient’s cardiac function and potential thrombosis in the heart and lower limb venous. rFVIIa was chosen as the safest RT option. Although expensive, rFVIIa is regarded as the overall best RT option,^[35]^ without introducing coagulation factors other than the defective, and is associated with effective hemostasis^[36]^ and low risk of thrombosis.^[28,32]^ The patient ultimately received a total of 8 doses of rFVIIa and 1 dose of FFP serving as the final dose at the end of postoperative recovery period. No other hemostatic drugs were used.

In conclusion, we presented a case of asymptomatic FVIID undergoing craniotomy for resection of brain tumor, where the hemostasis challenge was rare and stern in the context of asymptomatic FVIID. We suggest that in the case of asymptomatic patients undergoing surgical procedures with extremely high risk of bleeding, the individual pharmacodynamics of rFVIIa should be evaluated first. Furthermore, perioperative coagulation state, hemostasis and thrombosis events should be closely observed, and the interval and dosage of rFVIIa should be adjusted accordingly. These can effectively help prevent excessive surgical bleeding. The prevention of complicating venous thrombosis should also be noted after major surgery for patients with FVIID. An optimized strategy on perioperative management for such patients requires experience from more case studies and reports.

## Acknowledgments

We thank Fuyong Zhang and Yunhua Huang for data collection.

## Author contributions

**Conceptualization:** Chaoyu Huang, Wuning Mo, Faquan Lin.

**Data curation:** Chaoyu Huang, Yongjia Yu.

**Investigation:** Chaoyu Huang, Yongjia Yu, Ningneng Zhai.

**Supervision:** Faquan Lin.

**Writing – original draft:** Chaoyu Huang, Yongjia Yu, Ningneng Zhai.

**Writing – review & editing:** Chaoyu Huang, Wuning Mo, Faquan Lin.

## References

[1] Mumford AD Ackroyd S Alikhan R. Guideline for the diagnosis and management of the rare coagulation disorders: a United Kingdom Haemophilia Centre Doctors’ Organization guideline on behalf of the British Committee for Standards in Haematology. Br J Haematol. 2014;167:304–26. 25100430 10.1111/bjh.13058

[2] Mannucci PM Duga S Peyvandi F. Recessively inherited coagulation disorders. Blood. 2004;104:1243–52. 15138162 10.1182/blood-2004-02-0595

[3] Bernardi F Mariani G. Biochemical, molecular and clinical aspects of coagulation factor VII and its role in hemostasis and thrombosis. Haematologica. 2021;106:351–62. 33406812 10.3324/haematol.2020.248542 PMC7849579

[4] Herrmann FH Wulff K Auerswald G. Factor VII deficiency: clinical manifestation of 717 subjects from Europe and Latin America with mutations in the factor 7 gene. Haemophilia. 2009;15:267–80. 18976247 10.1111/j.1365-2516.2008.01910.x

[5] Napolitano M Siragusa S Mariani G. Factor VII deficiency: clinical phenotype, genotype and therapy. J Clin Med. 2017;6:38. 28350321 10.3390/jcm6040038 PMC5406770

[6] Lapecorella M Mariani G. Factor VII deficiency: defining the clinical picture and optimizing therapeutic options. Haemophilia. 2008;14:1170–5. 19141157 10.1111/j.1365-2516.2008.01844.x

[7] Mariani G Herrmann FH Dolce A. Clinical phenotypes and factor VII genotype in congenital factor VII deficiency. Thromb Haemost. 2005;93:481–7. 15735798 10.1160/TH04-10-0650

[8] Peyvandi F Palla R Menegatti M. Coagulation factor activity and clinical bleeding severity in rare bleeding disorders: results from the European Network of Rare Bleeding Disorders. J Thromb Haemost. 2012;10:615–21. 22321862 10.1111/j.1538-7836.2012.04653.x

[9] Mariani G Dolce A Napolitano M. Invasive procedures and minor surgery in factor VII deficiency. Haemophilia. 2012;18:e63–5. 22356641 10.1111/j.1365-2516.2012.02751.x

[10] Di Minno MND Napolitano M Dolce A. Role of clinical and laboratory parameters for treatment choice in patients with inherited FVII deficiency undergoing surgical procedures: evidence from the STER registry. Br J Haematol. 2018;180:563–70. 29235093 10.1111/bjh.15055

[11] Shapiro A. The use of prophylaxis in the treatment of rare bleeding disorders. Thromb Res. 2020;196:590–602. 31420204 10.1016/j.thromres.2019.07.014

[12] Sheth S Soff G Mitchell B. Managing incidentally diagnosed isolated factor VII deficiency perioperatively: a brief expert consensus report. Expert Rev Hematol. 2012;5:47–50. 22272705 10.1586/ehm.11.76

[13] Mariani G Dolce A Batorova A. Recombinant, activated factor VII for surgery in factor VII deficiency: a prospective evaluation - the surgical STER. Br J Haematol. 2011;152:340–6. 21158750 10.1111/j.1365-2141.2010.08287.x

[14] Benlakhal F Mura T Schved JF. A retrospective analysis of 157 surgical procedures performed without replacement therapy in 83 unrelated factor VII-deficient patients. J Thromb Haemost. 2011;9:1149–56. 21486425 10.1111/j.1538-7836.2011.04291.x

[15] Trossaert M Chamouard V Biron-Andreani C. Management of rare inherited bleeding disorders: proposals of the French Reference Centre on haemophilia and rare coagulation disorders. Eur J Haematol. 2023;110:584–601. 36748278 10.1111/ejh.13941

[16] Menegatti M Peyvandi F. Treatment of rare factor deficiencies other than hemophilia. Blood. 2019;133:415–24. 30559262 10.1182/blood-2018-06-820738

[17] Frattini F Bonfanti C Piccoli P. Triple coronary artery bypass graft surgery in a patient with factor VII deficiency: a case report. Haemophilia 2013;19:e268–9. 23647837 10.1111/hae.12164

[18] Livnat T Shenkman B Spectre G. Recombinant factor VIIa treatment for asymptomatic factor VII deficient patients going through major surgery. Blood Coagul Fibrinolysis. 2012;23:379–87. 22527290 10.1097/MBC.0b013e328352e8e2

[19] Khazi FM Siddiqi NR Karaly YM. Factor VII deficiency: do all need replacement for cardiac surgery? Asian Cardiovasc Thorac Ann. 2019;27:42–4. 30789010 10.1177/0218492317702784

[20] Windyga J Zbikowski P Ambroziak P. Management of factor VII-deficient patients undergoing joint surgeries--preliminary results of locally developed treatment regimen. Haemophilia. 2013;19:89–93. 22845882 10.1111/j.1365-2516.2012.02921.x

[21] Shirasawa H Yoshioka T Sawada K. Repeated recombinant activated factor VII administration in a patient with congenital factor VII deficiency undergoing modified radical hysterectomy: a case report. Haemophilia. 2014;20:e101–3. 24261688 10.1111/hae.12312

[22] Liu N Aldea S Francois D. Recombinant activated factor VII for a patient with factor VII deficiency undergoing urgent intracerebral haematoma evacuation with underlying cavernous angioma. Br J Anaesth. 2009;103:858–60. 19846405 10.1093/bja/aep293

[23] Yoshimura T Hayami S Kawai M. Perioperative management for patient with congenital factor VII deficiency who underwent laparoscopic cholecystectomy: case report. Int J Surg Case Rep. 2022;92:106892. 35278984 10.1016/j.ijscr.2022.106892 PMC8914216

[24] Brunori A Greco R Oddi G. Successful excision of hemorrhagic cavernous angioma in a patient with severe factor VII deficiency: perioperative treatment with factor VII concentrate. Neurosurg Rev. 1997;20:67–70. 9085292 10.1007/BF01390530

[25] Miyata N Isaka M Kojima H. Continuous infusion of recombinant activated factor VII for bleeding control after lobectomy in a patient with inherited factor VII deficiency. Gen Thorac Cardiovasc Surg. 2016;64:177–80. 25056454 10.1007/s11748-014-0455-1

[26] Gerlach R Krause M Seifert V. Hemostatic and hemorrhagic problems in neurosurgical patients. Acta Neurochir (Wien). 2009;151:873–900; discussion 900. 19557305 10.1007/s00701-009-0409-z

[27] Shi S Cheng J Chen H. Preoperative and intraoperative predictors of deep venous thrombosis in adult patients undergoing craniotomy for brain tumors: a Chinese single-center, retrospective study. Thromb Res. 2020;196:245–50. 32919179 10.1016/j.thromres.2020.09.005

[28] Napolitano M Dolce A Batorova A. Replacement therapy in inherited factor VII deficiency: occurrence of adverse events and relation with surgery. Haemophilia. 2015;21:e513–7. 26249164 10.1111/hae.12782

[29] Li L Wu X Wu W. A case-report of the unprovoked thrombotic event in a patient with thymoma and severe FVII deficiency. Thromb J. 2023;21:52. 37143073 10.1186/s12959-023-00494-3 PMC10157595

[30] Kang D Cha H Park SE. Paradoxical massive pulmonary thromboembolism in a postpartum woman with factor VII deficiency with bleeding tendency: a case report. Medicine (Baltim). 2023;102:e33437. 10.1097/MD.0000000000033437 PMC10082282 37026947

[31] Girolami A Tezza F Scandellari R. Associated prothrombotic conditions are probably responsible for the occurrence of thrombosis in almost all patients with congenital FVII deficiency critical review of the literature. J Thromb Thrombolysis. 2010;30:172–8. 20044773 10.1007/s11239-009-0435-y

[32] Rajpurkar M Croteau SE Boggio L. Thrombotic events with recombinant activated factor VII (rFVIIa) in approved indications are rare and associated with older age, cardiovascular disease, and concomitant use of activated prothrombin complex concentrates (aPCC). J Blood Med. 2019;10:335–40. 31572039 10.2147/JBM.S219573 PMC6757140

[33] Chen Y Hu F Wang J. Clinical features of craniopharyngioma with tumoral hemorrhage: a retrospective case-controlled study. Frontiers in Surgery. 2022;9:845273. 35360427 10.3389/fsurg.2022.845273 PMC8963871

[34] Chandrakasan S Sood S Ham S. Risk factors and management of deep venous thrombosis in children following post-surgical hypopituitarism in craniopharyngioma. Pediatr Blood Cancer. 2011;57:175–7. 21557464 10.1002/pbc.22937

[35] Mariani G Konkle BA Ingerslev J. Congenital factor VII deficiency: therapy with recombinant activated factor VII -- a critical appraisal. Haemophilia. 2006;12:19–27. 16409171 10.1111/j.1365-2516.2006.01180.x

[36] Mariani G Napolitano M Dolce A. Replacement therapy for bleeding episodes in factor VII deficiency: a prospective evaluation. Thromb Haemost. 2013;109:238–47. 23238632 10.1160/TH12-07-0476

